# Type of fitness cost influences the rate of evolution of resistance to transgenic Bt crops

**DOI:** 10.1111/1365-2664.12680

**Published:** 2016-05-05

**Authors:** Sean C. Hackett, Michael B. Bonsall

**Affiliations:** ^1^ Department of Zoology Mathematical Ecology Research Group University of Oxford South Parks Road Oxford OX1 3PS UK; ^2^ St. Peter's College New Inn Hall Street Oxford OX1 2DL UK

**Keywords:** *Bacillus thuringiensis*, density dependence, dynamic programming, evolution, high‐dose/refuge strategy, population dynamics, population genetics, resistance management, selection, transgenic crops

## Abstract

1. The evolution of resistance to pesticides by insect pests is a significant challenge for sustainable agriculture. For transgenic crops expressing *Bacillus thuringiensis* (Bt), crystalline (Cry) toxins resistance evolution may be delayed by the high‐dose/refuge strategy in which a non‐toxic refuge is planted to promote the survival of susceptible insects. The high‐dose/refuge strategy may interact with fitness costs associated with resistance alleles to further delay resistance. However, while a diverse range of fitness costs are reported in the field, they are typically represented as a fixed reduction in survival or viability which is insensitive to ecological conditions such as competition. Furthermore, the potential dynamic consequences of restricting susceptible insects to a refuge which represents only a fraction of the available space have rarely been considered.
2. We present a generalized discrete time model which utilizes dynamic programming methods to derive the optimal management decisions for the control of a theoretical insect pest population exposed to Bt crops. We consider three genotypes (susceptible homozygotes, resistant homozygotes and heterozygotes) and implement fitness costs of resistance to Bt toxins as either a decrease in the relative competitive ability of resistant insects or as a penalty on fecundity. Model analysis is repeated and contrasted for two types of density dependence: uniform density dependence which operates equally across the landscape and heterogeneous density dependence where the intensity of competition scales inversely with patch size and is determined separately for the refuge and Bt crop.
3. When the planting of Bt is decided optimally, fitness costs to fecundity allow for the planting of larger areas of Bt crops than equivalent fitness costs that reduce the competitive ability of resistant insects.
4. Heterogeneous competition only influenced model predictions when the proportional area of Bt planted in each season was decided optimally and resistance was not recessive.
5. *Synthesis and applications*. The high‐dose/refuge strategy alone is insufficient to preserve susceptibility to transgenic *Bacillus thuringiensis* (Bt) crops in the long term when constraints upon the evolution of resistance are not insurmountable. Fitness costs may enhance the delaying effect of the refuge, but the extent to which they do so depends upon how the cost is realized biologically. Fitness costs which apply independently of other variables may be more beneficial to resistance management than costs which are only visible to selection under a limited range of ecological conditions.

## Abstract 2

The high‐dose/refuge strategy alone is insufficient to preserve susceptibility to transgenic *Bacillus thuringiensis* (Bt) crops in the long term when constraints upon the evolution of resistance are not insurmountable. Fitness costs may enhance the delaying effect of the refuge, but the extent to which they do so depends upon how the cost is realized biologically. Fitness costs which apply independently of other variables may be more beneficial to resistance management than costs which are only visible to selection under a limited range of ecological conditions.

## Introduction

The evolution of resistance to pesticides and other population control measures by insect pests poses a significant challenge to both public health and agriculture. However, taking action to regulate or remove an insect population may introduce a selection pressure. This continuous selection pressure favours the survival of individuals expressing some level of tolerance to control, eroding our future capacity to manage the pest. As most recently commercialized insecticides are variants of previously isolated or synthesized compounds and developing and launching a new pesticide is estimated to take over a decade (REX Consortium 2013), it is unreasonably optimistic to assume that there will always be an alternative product available when control fails (Mitchell & Onstad 2014). Therefore, the sustained control of insect populations over prolonged periods requires that the strength of selection for resistance be constrained and/or reduced (REX Consortium 2013).

Recognition of the need to regulate selection for resistance has facilitated the development (and implementation) of the high‐dose/refuge strategy, particularly in conjunction with transgenic Bt crops (Tabashnik 2004). This approach uses simple population genetics to delay the evolution of resistance to the *Bacillus thuringiensis* (Bt)‐derived Cry toxins expressed within transgenic plants by providing a non‐toxic refuge to promote the survival of susceptible insects which then confer their susceptibility upon the next generation (Tabashnik 2004). The susceptible insects produced by the refuge can significantly delay the introgression of an initially rare resistance allele (Carrière & Tabashnik 2001; Tabashnik 2004; Carrière, Crowder & Tabashnik 2010). The introgression of resistance will be further delayed by the expression of a Bt toxin dose sufficient to reduce the survival of heterozygotes, rendering the resistant phenotype functionally recessive (Gould 1998; Tabashnik 2004). When resistance alleles are rare and resistance is functionally recessive, the combination of a high toxin dosage and a refuge significantly constrains the rate of resistance evolution (Carrière & Tabashnik 2001; Tabashnik 2004; Alphey *et al*. 2008). The high‐dose/refuge strategy has been successful in maintaining the susceptibility of agricultural insect pests to transgenic Bt crops with failures generally being linked to either a deviation from prescribed refuge sizes or the expression of an insufficient toxin dose by the transgenic plants (Tabashnik, Brévault & Carrière 2013; García *et al*. 2015).

In spite of the strengths of the high‐dose/refuge strategy, less understood threats to the efficacy of this approach are ecological effects such as intraspecific (within species) competition between insects within the refuge (Onstad, Shelton & Flexner 2014). Competition is predicted to accelerate the evolution of resistance when carrying capacities are low or density dependence is overcompensating. These conditions may be facilitated by the spatial subdivision imposed upon the landscape by the high‐dose/refuge strategy (Onstad, Shelton & Flexner 2014). Superficially, we would not anticipate insects in a managed field to be able to attain numbers such that their density became self‐limiting without also losing the majority of the available yield. However, confining susceptible insects to comparatively small areas may permit negative density‐dependent effects at lower densities than would otherwise be predicted. Theoretical work has suggested that competition between susceptible insects may undermine the delaying effect of the refuge by reducing the production of susceptible adults (Sisterson, Antilla & Carrière 2004; Glaum, Ives & Andow 2011).

An additional consideration is the potential synergy between the high‐dose/refuge strategy and any fitness costs associated with resistance alleles (Tabashnik 2004; Alphey *et al*. 2008). Assuming a high toxin dose, resistance alleles of small phenotypic effect will be selected against; only alleles conferring sufficient tolerance to overcome the expressed toxin dose will be favoured. Such tolerance is most likely to be conferred by a major mutation that may also have pleiotropic effects which negatively influence other processes pertinent to survival and reproduction (Macnair 1991; Coustau, Chevillon & ffrench‐Constant 2000; Gassmann, Onstad & Pittendrigh 2009). Thus, resistant insects will likely be subject to fitness costs which may constrain the introgression of the resistance allele (Carrière & Tabashnik 2001; Tabashnik 2004; Alphey *et al*. 2008). However, the degree to which fitness costs delay resistance and synergize with a high‐dose/refuge approach is contingent upon the genetic dominance of the resistance allele (Carrière & Tabashnik 2001; Tabashnik 2004; Carrière, Crowder & Tabashnik 2010). The dominance of resistance determines the extent to which the resistance of an insect with a single copy of the allele (a heterozygote) compares to that of an insect with two copies of the allele (a homozygote). If the resistance of a heterozygote is comparable to that of a homozygote for a given toxin dosage, then resistance is dominant. Dominance spans a spectrum from incomplete dominance (the heterozygote cannot tolerate toxin exposure to the same extent as a homozygote) to complete dominance (the heterozygote is functionally identical to a homozygote). If the heterozygote exhibits no resistance, then resistance is termed recessive, while if it is intermediate between each homozygote, resistance is additive. Intuitively, recessive resistance is simpler to manage than dominant resistance.

A range of fitness costs have been reported, from both laboratory and field, for insects expressing resistance to chemical control agents (Gassmann *et al*. 2009). However, fitness costs explicitly associated with resistance to Bt crops remain poorly understood (Jakka, Knight & Jurat‐Fuentes 2014). This shortfall in available data can be partly attributed to the success of the high‐dose/refuge strategy; if instances of field‐evolved resistance are rare, so studies of field‐evolved resistance to Bt crops are similarly sparse. Recent efforts to categorize and quantify fitness costs associated with field‐evolved resistance to Bt crops have reported variable results (Jakka, Knight & Jurat‐Fuentes 2014; Dangal & Huang 2015; García *et al*. 2015; Ingber & Gassmann 2015).

Difficulties in the consistent identification of fitness costs may arise from the simplifying assumption that fitness costs are constant. However, fitness is a complex function of many factors influencing both survival and reproduction. Some of these factors will be ecological and may exaggerate or conceal pleiotropic effects associated with a resistance allele (Gassmann, Onstad & Pittendrigh 2009); that is, fitness costs may exhibit a degree of context dependence and may increase or only become visible to selection in the presence of additional environmental stressors. For example, fitness costs may be exaggerated by factors such as host plant variety (Bird & Akhurst 2007), parasites and pathogens (Raymond *et al*. 2007), temperature (Zhang *et al*. 2015) or biotic interactions (Becker & Liess 2015). When fitness costs are context sensitive, the resistance allele frequency may not necessarily decline in the absence of selection. Unless the context is appropriate, the resistance allele may behave as if it were cost‐free and resistance will evolve more rapidly (Jakka, Knight & Jurat‐Fuentes 2014; García *et al*. 2015; Ingber & Gassmann 2015).

Here, we develop a generalized discrete time model which utilizes dynamic programming methods to derive optimal management decisions for the control of a theoretical pest population. This approach allows us to represent managers as non‐static, goal‐orientated entities that adjust their decisions over time based upon the information available to them. In addition, dynamic programming captures the discrete time nature of pest management decisions. We consider a single resistance gene segregating at a diallelic locus and implement fitness costs as either a decrease in the relative competitive ability of resistant insects or as a penalty to fecundity. Costs to fecundity apply irrespective of additional factors while a decrease in competitive ability only penalizes resistant insects when the total population density is sufficiently large. We find that costs to fecundity allow for the selection of larger areas of Bt crops than costs on competitive ability. This implies that the mechanistic effect of fitness costs in the field is a significant consideration in the development of resistance management strategies.

## Materials and methods

We consider the management of a hypothetical univoltine insect pest population feeding in a closed landscape with no alternative hosts. The manager seeks to suppress the pest population by selecting the area of the landscape which is to be planted with transgenic plants expressing insecticidal Bt toxins. Thus, the manager's decisions divide the landscape into two distinct connected patches of variable magnitude: the Bt crop and the refuge. The proportion of the landscape allocated to toxic plants is denoted φ and so the proportional area of the refuge is 1 − φ. The manager specifies the value of φ each season such that the cumulative pest burden experienced over the time horizon, *T*, is minimized. It is in the manager's interest to control resistance; therefore, we assume that the manager attempts to conserve the future utility of the transgenic crops by actively seeking to maintain the frequency of the resistance allele below some critical threshold. The model has two distinct components: a dynamic programming model which identifies the best decision available to the manager for a given set of conditions and a population submodel characterizing the dynamics and genetics of the pest population.

### Pest Submodel

The growth of the pest population is described by a density‐dependent selection model (Roughgarden 1971) which uses the two‐parameter density dependence function developed by Maynard Smith & Slatkin (1973). This function was selected for its capacity to describe a range of dynamic behaviours (Bellows 1981). An insect may belong to one of three alternative genotypes: susceptible homozygotes (*ss*), resistant heterozygotes (*sr*) or resistant homozygotes (*rr*). The frequency of the susceptible allele, *s*, is denoted *p* and the corresponding frequency of the resistance allele, *r*, is denoted *q*. The pest submodel proceeds as follows:

1. Pests are divided between the refuge and toxic patch (where φ > 0) in a ratio proportional to patch size.
2. Pests within the Bt patch are exposed to toxins and mortality is quantified.
3. Pest mortality via intraspecific competition operates either across the entire space or separately within each patch.
4. Surviving larvae mature and mate at random to generate the next generation.

Larvae emerge into a landscape which, contingent upon the decision taken by the manager at the beginning of the season, may be a single contiguous refuge or, more probably, is subdivided into a toxic patch and a non‐toxic refuge. We assume uniform oviposition so, of *N*_*t*_ larvae hatching during season *t*, φ*N*_*t*_ will develop in the toxic patch and (1 − φ)*N*_*t*_ will be localized within the refuge. Larvae remain within their natal patch until maturation.

On hatching, larvae commence feeding and the φ*N*_*t*_ larvae within the toxic patch are exposed to Bt. The proportion of larvae that survive exposure to Bt is contingent upon their genotype, *g*, and is denoted *S*_*g*_ where *S*_*ss*_ ≤ *S*_*sr*_ ≤ *S*_*rr*_. The total population density subsequent to toxin exposure is denoted $N_{t}^{\prime }$ which is the sum of the refuge population and the survivors within the Bt patch. Larvae that survive toxin exposure (or were not exposed) undergo intraspecific competition. Two different implementations of competition are considered. In the first, density‐dependent mortality acts uniformly, the entire landscape is treated as a single whole and differences in pest abundance between patches are ignored. However, given that there is no larval movement and thus competition will vary locally, this could underestimate the impact of competition when the population within a patch is large relative to its size. The second, heterogeneous, implementation accounts for this by specifying an inverse relationship between competitive mortality and patch size. Density dependence is then evaluated separately for each patch. To accommodate both representations, we derive the number of larvae of genotype *g* in patch *i*, $N_{t,g,i}^{\prime \prime }$, surviving competition and progressing into the reproductive phase as:

(eqn 1) $$N_{t,g,i}^{\prime \prime }=\frac{N_{t,g,i}^{\prime }}{1+{\left(\frac{\alpha_{g}}{m_{i}}N_{t,i}^{\prime }\right)}^{\beta }}$$

where α_*g*_ and β are the parameters of Maynard Smith & Slatkin (1973). The value of β determines the intensity of competition and is assumed to be independent of genotype. Values of β ≈ 1 are suggestive of undercompensating density dependence, while β > 1 shifts the dynamics towards increasingly intense, overcompensating, competition (Bellows 1981). The value of α_*g*_ (0 < α_*g*_) determines the per capita sensitivity of insects of genotype *g* to intraspecific competition and sets the threshold population density beyond which the net population growth rate for genotype *g* becomes negative. To link competition and patch size, we scale the value of α_*g*_ by the factor $\frac{1}{m_{i}}$ where *m*_*i*_ is the relative area of patch *i* (i.e. φ for the toxic patch and 1 − φ for the refuge). Thus, the sensitivity to competition of insects within a patch increases as patch size declines. If the field has been planted with only one type of crop or competition is specified as uniform, then the subscript *i* is unnecessary and *m* = 1 for the sole patch.

Insects carrying resistance alleles may be subject to fitness costs. Fitness costs were implemented as either an increase in the value of α_*g*_ (so that α_*ss*_ ≤ α_*sr*_ ≤ α_*rr*_) or a decrease in fecundity, λ_*g*_. Costs to competition may be conceptualized as a decrease in the efficiency with which resistant insects acquire resources, or an increase in their resource requirement relative to susceptible insects. Costs to fecundity represent a reduction in the efficiency with which resistant insects convert acquired resources into offspring. While the mechanism differs, both costs constrain the maximum size a population of resistant insects can attain for given space and resources. Where costs increase α_*g*_, resistant insects will experience greater levels of mortality at high population densities than susceptible insects. As density dependence is not selectively neutral when competitive fitness costs apply, allele frequencies must be recalculated prior to reproduction:

(eqn 2) $$q_{t}^{\prime }=\frac{N_{t,rr}^{\prime }+\frac{1}{2}N_{t,sr}^{\prime }}{N_{t}^{\prime }}$$

(eqn 3) $$p_{t}^{\prime }=1-q_{t}^{\prime }$$

Insects which survive intraspecific competition then mature. Mating is at random with respect to both genotype and space. The number of insects in the next generation is as follows:

(eqn 4) $$N_{t+1}=N_{t}^{\prime \prime }\left(p^{\prime 2}\lambda_{ss}+2p^{\prime }q^{\prime }\lambda_{sr}+q^{\prime 2}\lambda_{rr}\right)$$

where $N_{t}^{\prime \prime }$ is the total number of insects in the post‐competition population. The value of λ_*g*_ denotes the average fecundity of an insect of genotype *g*. When resistance does not influence fecundity λ_*ss*_ = λ_*sr*_ = λ_*rr*_ and the intergenerational change in the number of insects simplifies to $N_{t+1}=\lambda N_{t}^{\prime \prime }$. The resistance allele frequency in the next generation is as follows:

(eqn 5) $$q_{t+1}=\frac{q^{\prime 2}\lambda_{rr}+p^{\prime }q^{\prime }\lambda_{sr}}{p^{\prime 2}\lambda_{ss}+2p^{\prime }q^{\prime }\lambda_{sr}+q^{\prime 2}\lambda_{rr}}$$

Fitness cost magnitude is specified using the parameter *c*, where 0 ≤ *c* ≤ 1, to represent the proportional decrease or increase in the value of the relevant parameter. That is, fitness costs are implemented for the resistant homozygote as:

(eqn 6) $$\alpha_{rr}=\left(1+c\right)\alpha_{ss}$$

(eqn 7) $$\lambda_{rr}=\left(1-c\right)\lambda_{ss}$$

Only a single fitness cost is considered in a given simulation (that is, penalties are applied to either fecundity or competition but never both). The dominance of resistance and its correlated fitness costs are determined by the value of the parameter *h* where 0 ≤ *h* ≤ 1. A value of *h* = 0 denotes fully recessive resistance (a single copy of the *r* allele has no effect). As *h* tends to 1, resistance becomes increasingly dominant with *h* = 1 indicating fully dominant resistance (a single copy of the *r* allele is equivalent to two copies). The values of *S*_*sr*_, α_*sr*_ and λ_*sr*_ are calculated as follows:

(eqn 8) $$\left(1-h\right)x_{ss}+hx_{rr}$$

where *x* represents the parameter of interest.

Values for the heterozygote then depend on the dominance of the resistance allele. This assumes that the heritability of resistance and any correlated fitness costs are identical, but this need not be true (Gould 1998). To relax this assumption, unique heritability coefficients are assigned to *S*_*rr*_ and either α_*rr*_ or λ_*rr*_ depending on the fitness cost of interest. In this instance, let *h*_*res*_ denote the heritability of *S*_*rr*_ and *h*_*cost*_ represent the heritability of either α_*rr*_ or λ_*rr*_. For reference, the use of *h* without a subscript refers to instances where the heritability of resistance and fitness costs are identical (*h*_*res*_ = *h*_*cost*_).

### Decision‐Making

The decision process is captured using dynamic programming. We consider a manager seeking to suppress an insect pest and in doing so implicitly minimize yield loss over the considered time horizon. We acknowledge that this is a simplified interpretation of grower behaviour that excludes factors such as discounting or the likelihood that refuge plants will incur high levels of feeding damage relative to Bt plants. Such factors would bias decisions towards short‐term benefits and favour the more rapid depletion of susceptibility to Bt.

From the manager's perspective, resistance management is a means to an end which prolongs the efficacy of available control mechanisms. However, in the absence of control mechanisms which can reduce pest density, *N*, independently of the resistance allele frequency, *q*, the minimization of both pest density and resistance are, to an extent, mutually exclusive outcomes. Planting mostly transgenic crops strongly suppresses population density when resistance is rare but drives selection for resistance, decreasing the crops' control efficacy in subsequent seasons. Conversely, minimal plantings of Bt crops retain a greater number of susceptible alleles but enable greater pest population densities. A simple method for capturing this trade‐off in the decision model is to only consider decisions for which the *r* allele frequency is held below a specified critical threshold value, *q*_*c*_.

The area of the landscape to be planted with transgenic crops, φ, is selected from the control set $\Phi =\left\{\varphi_{i}\right\};$ in our investigations, we permitted fractions of the landscape ranging from 0 to 1 in increments of 0·1. This is chosen for each decision period, *t*, within the specified time horizon, *T*, such that the cumulative pest burden experienced between period *t* and *T* is minimized subject to the constraint that the resistance allele does not attain a frequency greater than *q*_*c*_. This objective is captured in a dynamic programming equation which calculates the value, *V*, (measured as the post‐reproductive pest density at the end of the period) of selecting control φ_*i*_ during time period *t* for a manager who began the period with a total pest population density of *N*_*t*_ as:

(eqn 9) $$V_{i}\left(N_{t},t\right)=\left(N_{t+1}+F\left(N_{t+1},t+1\right)\right)|q_{t+1}<q_{c}$$

The cumulative pest burden endured by a manager who selects control φ_*i*_ in period *t* and behaves optimally from then onwards is represented by *F*(*N*_*t*+1_, *t* + 1). The current optimal decision is that which produces the minimum value of *V*:

(eqn 10) $$F\left(N_{t},t\right)=\min_{\varphi_{i}}V.$$

For simplicity, we assume that the manager accrues no additional benefit beyond the terminal time period, *T*, and thus is not concerned with management of the pest beyond that. This allows us to state the terminal condition:

(eqn 11) $$F\left(N_{T},T\right)=0$$

for all possible values of *N* (Clark & Mangel 2000). The optimal solution to this problem is derived numerically by a dynamic programming algorithm with two state variables, *N* and *q*. The state variables are presented within the algorithm as discretized vectors, ***N*** and ***q***, which have *n* and *l* divisions between their least and greatest values, respectively. The algorithm derives values for $F\left(N_{t},t\right)$ from *F*(*N*_*t*+1_, *t* + 1) for each combination *N* and *q* via backwards iteration. Where the state values produced by a control decision are not found within their corresponding state vector, linear interpolation is used to identify the best decision (Clark & Mangel 2000).

### Model Exploration

#### Fixed landscape partitioning

All simulations were carried out in r version 3.13. We begin by simulating the dynamics of the pest submodel independently of the decision model to provide baseline estimates for the time required for resistance to evolve when the landscape partitioning is static. For all simulations, we assume the per capita sensitivity of susceptible homozygotes to competition to be α_*ss*_ = 1 × 10^−6^ with average fecundity λ_*ss*_ = 2. No susceptible homozygotes survive exposure to Bt toxins (*S*_*ss*_ = 0) and resistance is complete (*S*_*rr*_ = 1). Where the value of the parameter β was varied, it was restricted to values between 1 and 3. The evolution of resistance was simulated over 100 generations for two fixed plantings of Bt, φ = (0·95, 0·8). The influence of dominance and fitness costs upon resistance evolution was considered for both types of density dependence. Pest populations were initiated with *N*_0_ = 1000 insects and an initial resistance allele frequency of *q*_0_ = 0·01.

#### Dynamic partitioning

**Figure 1:**
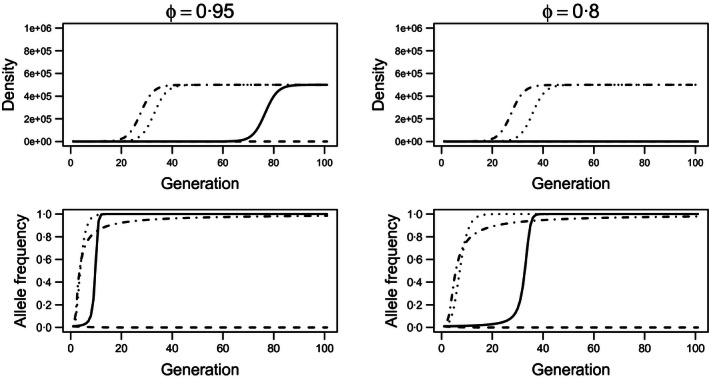
Population size (top row) and resistance allele frequency (bottom row) over 100 generations for landscapes planted with 95% (left column) and 80% (right column) Bt, respectively, when resistance is associated with a 25% reduction in fecundity (λ_*rr*_ = 0·75λ_*ss*_). Density dependence is undercompensating and uniform (β = 1). Solid lines show recessive resistance with a recessive fitness cost (*h*_*res*_ = 0, *h*_*cost*_ = 0). Dashed lines depict recessive resistance with a dominant fitness cost (*h*_*res*_ = 0, *h*_*cost*_ = 1). Dotted lines refer to additive resistance (codominant) with a dominant fitness cost (*h*_*res*_ = 0·5, *h*_*cost*_ = 1), and dash‐dot lines illustrate dominant resistance with a dominant fitness cost (*h*_*res*_ = 1, *h*_*cost*_ = 1). Populations were founded with *N*_0_ = 1000 insects and initial resistance allele frequency *q*_0_ = 0·01.

In deriving the numerical solution for the decision model, the algorithm compiles a decision array containing the best permissible decision for each combination of state values within each time period. We used state vectors of length *n* = 20 and *l* = 11 which assumed values in the ranges 1 ≤ *n* ≤ 1 000 000 and 0 ≤ *l* ≤ 1, respectively. These predictions were evaluated via forwards iteration (Clark & Mangel 2000). Beginning with an initial population density of *N*_0_ = 1000 insects and an initial *r* allele frequency of *q*_0_ = 0·01, the intergenerational change in both *N* and *q* was simulated from *t* = 0 to *t* = *T*. Given that the resistance allele exceeded the critical threshold of *q*_*c*_ = 0·5 within 20–30 generations for fixed patch sizes (e.g. Figs 1, S1, S2 & S3 in Supporting Information), the time horizon for the decision model was set at *T* = 60 generations. At the beginning of each time period, the element of the decision array which corresponds to the optimal decision was identified (using linear interpolation as appropriate) and implemented. Simulations were run for both uniform and heterogeneous density dependence over 20 nonzero values of each fitness cost and average Bt usage was recorded for each cost value. The proportional decrease in the fecundity of resistant homozygotes was lowered from λ_*rr*_ = λ_*ss*_ (no reduction in fecundity) to λ_*rr*_ = 0·7λ_*ss*_ (a 30% reduction) in increments of 0·015. The proportional increase in the sensitivity of the resistant homozygote to competition was increased from α_*rr*_ = α_*ss*_ to α_*rr*_ = 2α_*ss*_ (resistant homozygotes are twice as sensitive to competition as susceptible homozygotes) in increments of 0·05. Results of these simulations are reported for three dominance scenarios: recessive resistance (*h* = 0), additive resistance (*h* = 0·5) and additive resistance with a dominant fitness cost (*h*_*res*_ = 0·5, *h*_*cost*_ = 1). Time series of model behaviour and state dynamics were also generated for fixed fitness cost intensities and three levels of dominance: *h* = 0·05 (weakly dominant), *h* = 0·5 (additive) and *h* = 1 (dominant).

## Results

### Fixed Landscape Partitioning

For a fixed partitioning of space between the refuge and the Bt crop, the high‐dose/refuge strategy delays resistance evolution and suppresses the pest population when resistance is fully recessive with larger refuges providing greater delays (Figs 1 and S1). Non‐recessive resistance (*h*_*res*_ > 0) reduces the efficacy of refuges and accelerates resistance evolution. The type of density dependence (uniform or heterogeneous) influenced neither resistance evolution nor the growth of the pest population (Fig. S1). Increasing the intensity of competition (β > 1) has a negligible influence upon population growth and resistance evolution. Neither fecundity nor competition costs strongly synergized with the high‐dose/refuge strategy for the simulated refuge sizes (Figs 1 and S2); high levels of toxin mortality alleviate competition and mask fitness costs even when the initial population density is large (Fig. S3). However, dominant fecundity costs strongly delayed resistance when the resistant phenotype was recessive (Fig. 1, dashed lines). Additionally, when both resistance and fecundity costs were recessive, the growth of the population was significantly slowed for larger refuge sizes (e.g. Fig. 1, φ = 0·8, top row, Fig. S4).

### Fitness Costs and Average Bt Usage

**Figure 2:**
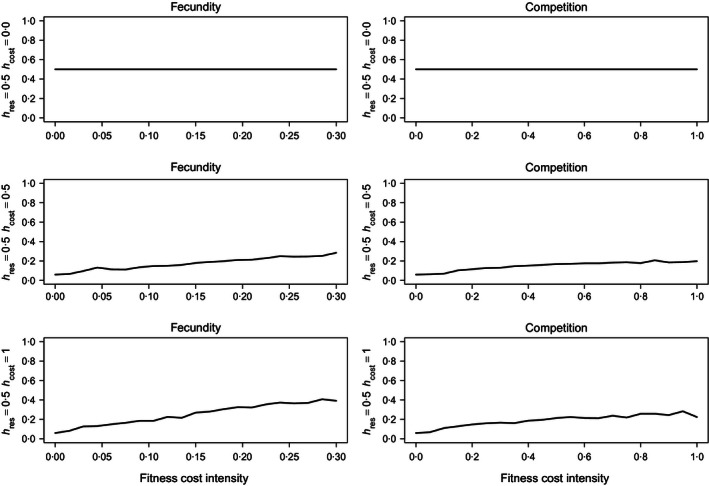
The mean proportion of the landscape allocated to Bt crop over *T* = 60 generations plotted against twenty nonzero levels of fitness costs to fecundity (λ_*g*_, left column) or competitive ability (α_*g*_,  right column) for a hypothetical pest population. Density dependence is undercompensating (β = 1) and uniform. Plots show (top to bottom): fully recessive resistance (*h*_*res*_ = 0, *h*_*cost*_ = 0), additive resistance (*h*_*res*_ = 0·5, *h*_*cost*_ = 0·5) and additive resistance with a dominant fitness cost (*h*_*res*_ = 0·5, *h*_*cost*_ = 1). The values on the *x*‐axis describe either the proportional decrease in the value of λ_*rr*_ relative to λ_*ss*_ (left column) or the proportional increase in the value of α_*rr*_ relative to α_*ss*_ (right column). *N*_0_ = 1000 and *q*_0_ = 0·01.

Fully recessive resistance may be controlled for the full extent of the considered time horizon by the continuous planting of Bt crops at a fixed level (Fig. 2, row 1). This result is insensitive to the type or severity of fitness cost, the type of density dependence and the intensity of competition (not shown). The level of Bt used is sensitive to the fecundity of the susceptible homozygotes and heterozygotes (not shown). The more rapidly the susceptible homozygote (which comprises the bulk of the founding population) reproduces relative to heterozygotes and resistant homozygotes, the greater the average level of Bt which may be planted while still holding the resistance allele beneath the critical threshold of *q*_*c*_ = 0·5.

When resistance is non‐recessive and carries fitness costs, the mean level of Bt selected by the decision model is sensitive to the magnitude of the cost (Fig. 2). Greater fitness costs promote larger optimal areas of Bt with the largest plantings being observed when fitness costs reduce fecundity (Fig. 2). High levels of toxin mortality when Bt patches are large accelerate the introgression of resistance and alleviate competition. Thus, high susceptible mortality rates in large areas of Bt reduce the impact of competition costs as the insect population must be large for these costs to impede resistance evolution. In contrast, a penalty to fecundity delays the introgression of resistance even when Bt crops are prevalent by constraining the number of offspring a resistant insect may produce. However, even large penalties to fecundity are insufficient to permit a return to the average levels of Bt crops observed for recessive resistance.

### Density Dependence, Fitness Costs and Time Series

When resistance is costless, with an initial population of *N*_0_ = 1000 insects and an initial resistance allele frequency of *q*_0_ = 0·01, fully recessive resistance (*h* = 0) is always treated with a fixed level of Bt as predicted by Fig. 2. Weakly dominant, cost‐free resistance (*h* = 0·05) may still be controlled but requires that Bt usage vary with time resulting in smaller average Bt areas (Figs 2 and S5). Further increases in the heritability of resistance leads to Bt usage declining to zero within 20 generations. Thus, average Bt usage against non‐recessive cost‐free resistance is low (Fig. 2).

**Figure 3:**
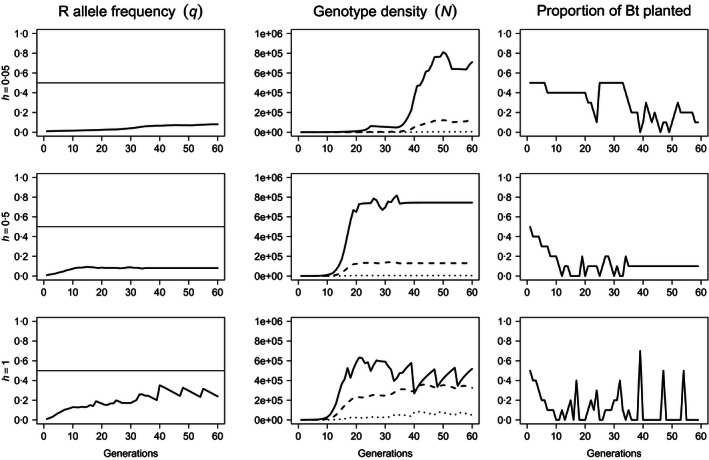
Resistance allele frequency (left‐most column), genotype abundance (central column) and proportional allocation of space to Bt crops (rightmost column) over *T* = 60 generations when resistance is associated with a 25% reduction in competitive ability (α_*rr*_ = 1·25α_*ss*_). Density dependence is undercompensating (β = 1) and uniform. The dominance of resistance was increased from *h* = 0·05 (top row), to *h* = 0·5 (centre row) and *h* = 1 (bottom row). The solid horizontal lines (left) are the critical allele frequency, *q*_*c*_ = 0·5. On plots of genotype‐specific density, solid lines represent susceptible homozygotes (*ss*), dashed lines represent heterozygotes (*sr*) and dotted lines represent resistant homozygotes (*rr*). *N*_0_ =  1000 and *q*_0_ = 0·01.

The existence of fitness costs offers the decision model additional flexibility. Time series for the resistance allele frequency, genotype abundance and Bt usage over *T* = 60 generations for a pest population with uniform density dependence and a fitness cost that reduces the competitive ability of resistant insects, α_*rr*_, by 25% relative to susceptible insects (α_*rr*_ = 1·25α_*ss*_) are shown in Fig. 3. Inheritance of resistance and fitness cost are assumed to be identical, and three levels of dominance are considered: weakly dominant (*h* = 0·05 *i*.*e*. near recessive), additive (*h* = 0·5) and dominant (*h* = 1). Decision trajectories for fully recessive resistance are unresponsive to fitness costs (not shown). As with cost‐free resistance, the pattern of Bt usage is principally determined by the dominance of resistance. The response to weakly dominant resistance is equivalent to that for cost‐free resistance (Fig. S5). When resistance is additive or dominant, Bt usage initially declines, as for cost‐free resistance. However, the competitive penalty renders the continued planting of Bt optimal either at a fixed low level (Fig. 3, middle row, *h* = 0·5) or at higher levels in acute periods interspersed with multiple consecutive generations in which Bt plants are absent (Fig. 3, bottom row, *h* = 1).

**Figure 4:**
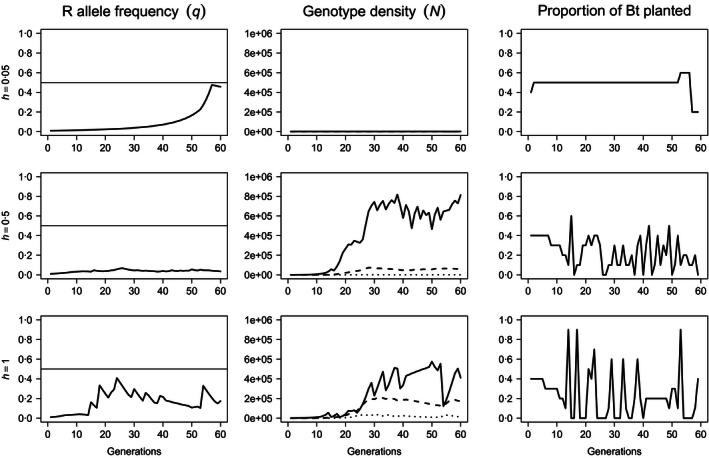
Resistance allele frequency (left‐most column), genotype abundance (central column) and proportional allocation of space to Bt crops (right‐most column) over *T* = 60 generations when resistance is associated with a 25% reduction in fecundity (λ_*rr*_ = 0·75λ_*ss*_). Density dependence is undercompensating (β = 1) and uniform. The dominance of resistance was increased from *h* = 0·05 (top row), to *h* = 0·5 (centre row) and *h* = 1 (bottom row). The solid horizontal lines (left) are the critical allele frequency, *q*_*c*_ = 0·5. On plots of genotype‐specific density, solid lines represent susceptible homozygotes (*ss*), dashed lines represent heterozygotes (*sr*) and dotted lines represent resistant homozygotes (*rr*). *N*_0_ = 1000 and *q*_0_ = 0·01.

Fitness costs on fecundity have a more pronounced effect upon the decision trajectories than competition costs of equivalent magnitude (Fig. 4). The impact of fecundity costs is sufficiently great that the strategy adopted to manage weakly dominant resistance (*h* = 0·05) is almost equivalent to that observed for fully recessive resistance (*cf*. Fig. 4, top row and Fig. 2, top row). Thus, fecundity costs influence the pest population even when expressed weakly. For additive (*h* = 0·5) and dominant (*h* = 1) resistance, moderate‐to‐large areas of Bt are planted more frequently than when fitness costs impaired competitive ability. However, Bt‐free recovery periods are still necessary to maintain the resistance allele frequency beneath the critical threshold (*q*_*c*_) and are most frequent for dominant resistance (Fig. 4, bottom row).

**Figure 5:**
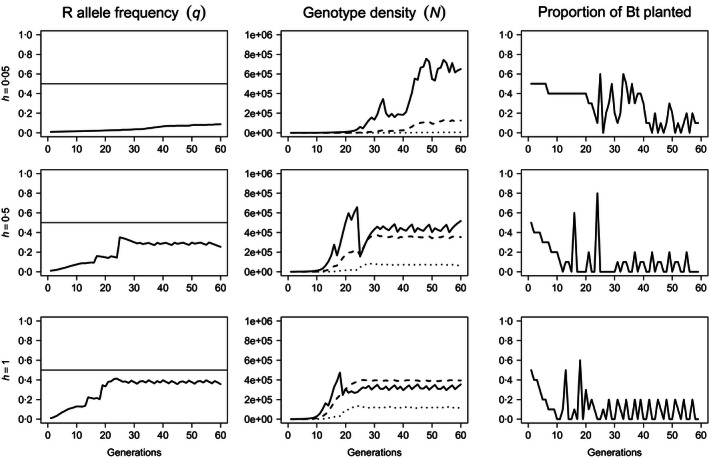
Resistance allele frequency (left‐most column), genotype abundance (central column) and proportional allocation of space to Bt crops (right‐most column) over *T* = 60 generations when resistance is associated with a 25% reduction in competitive ability (α_*rr*_ = 1·25α_*ss*_). Density dependence is undercompensating (β = 1) and heterogeneous. The dominance of resistance was increased from *h* = 0·05 (top row), to *h* = 0·5 (centre row) and *h* = 1 (bottom row). The solid horizontal lines (left) are the critical allele frequency, *q*_*c*_ = 0·5. On plots of genotype‐specific density, solid lines represent susceptible homozygotes (*ss*), dashed lines represent heterozygotes (*sr*) and dotted lines represent resistant homozygotes (*rr*). *N*_0_ =  1000 and *q*_0_ = 0·01.

While heterogeneous density dependence does not influence average Bt usage (not shown), it does influence the decision trajectories (illustrated in Fig. 5, with parameters as in Fig. 3). Heterogeneous density‐dependent mortality frequently replaces periods of static Bt levels with oscillations between larger and smaller areas of Bt crops. Similar effects are also observed for cost‐free resistance (Fig. S6) and fecundity costs (Fig. S7). In spite of these fluctuations, the general trends observed for heterogeneous density dependence remain similar to those for uniform density dependence.

Thus, the most significant constraint upon model behaviour is the dominance of the resistant phenotype, with greater dominance decreasing Bt usage. For a given level of dominance of resistance, the type and dominance of any fitness costs may relax these constraints, allowing for larger areas of Bt crop than would otherwise be optimal. Nested within this, the type of density dependence may alter the transient dynamics of the decision set. These findings are qualitatively summarized in Table 1.

**Table 1:** Summary of the general effect of the genetic dominance of resistance, fitness costs, the type of fitness cost and heterogeneous density dependence on the planting of Bt crops by the decision model. Downward pointing arrows indicate a reduction in the planting of Bt. Upward pointing arrows indicate an increase in the planting of Bt. Arrow thickness is indicative of the strength of the effect with thicker arrows representing stronger effects. Factors may interact; for example, dominant fecundity costs provide greater increases in Bt usage than dominant competition costs. Heterogeneity in competitive mortality between the refuge and Bt crop does not change average model behaviour but promotes oscillations in Bt usage which is indicated by the wave

Factor	Influence on planting of Bt	Explanation
Dominance of resistance	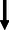	High heterozygote survival when resistance is dominant requires large refuges
Fitness costs on fecundity (λ)	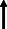	Fecundity costs delay resistance independently of other factors. They have a positive effect on Bt planting
Fitness costs on competitive ability (α)	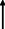	Competition costs delay resistance when the population is large. They increase Bt planting less effectively than fecundity costs
Dominance of fitness cost	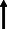	Increasing the dominance of either fitness cost promotes planting of Bt. The extent of the increase is determined by the type and dominance of the cost
Heterogeneous density dependence	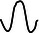	Heterogeneous density dependence promotes oscillating levels of Bt crops to correct for additional competitive mortality of susceptible insects

## Discussion

We used a decision model to explore the implications of different types of both fitness costs and density dependence on the management of resistance in a theoretical insect pest population feeding on an insecticidal Bt crop. We found that the heritability of resistance and any associated fitness costs had the greatest effect upon model decisions (e.g. Figs 3 and 4), while the type of density dependence only became relevant under particular conditions (non‐recessive resistance with non‐recessive costs, Fig. 5). These predictions indicate that differences between susceptible and resistant insects may not necessarily translate into management benefits. Thus, long‐term resistance management using the high‐dose/refuge strategy will benefit from additional control measures which are less sensitive to the heritability and pleiotropic effects of resistance genes.

The dominance of the resistant phenotype was the most significant determinant of model behaviour, significantly constraining the usage of Bt crops. This is congruent with the long established sensitivity of the high‐dose/refuge strategy to the dominance of resistance genes (Carrière & Tabashnik 2001; Tabashnik 2004; Carrière, Crowder & Tabashnik 2010). The phenotype of heterozygotes is crucial to the spread of rare alleles; high heterozygote survival rates require large refuges to maintain susceptibility. Notably, planting some Bt remained optimal even when cost‐free resistance was additive (*h* = 0·5) or dominant (*h* = 1), but this could only be justified for a few generations before the optimal decision was to plant the entire landscape as refuge (Fig. S5), indicating the failure of Bt crops. In contrast, fully recessive resistance (*h* = 0) was managed by planting Bt patches of fixed magnitude (Fig. 2, top row). These fixed values of Bt reflected the trade‐off between long‐ and short‐term killing required for sustainable resistance management and were sensitive to the fecundity of the susceptible homozygote, the principal driver of population growth when the resistance allele is rare. In effect, the model behaves as if susceptibility is a renewable resource (Mitchell & Onstad 2014) and allocates space between the refuge and Bt crop in a manner that regulates the number of susceptible insects while maintaining the resistance allele beneath the critical threshold. The more rapidly susceptible insects are replaced, the more that can be exposed to Bt in a given generation without prompting rapid selection for resistance. Results for dominant resistance may also be understood in this context. When resistance is dominant, the rate at which susceptibility is depleted is accelerated, narrowing the range of optimal Bt values, which indicates that refuges alone are insufficient to control dominant resistance.

Neither type of fitness cost significantly delays the evolution of resistance under constant selection (Figs 1 and S2), but both types of cost relaxed constraints upon the decision model. However, for a given level of dominance of resistance, costs to fecundity enabled greater average Bt usage than competition costs (e.g. Figs 2, 3, 4). Costs to fecundity most closely resemble the “few genes of large effect” classically associated with resistance evolution (Macnair 1991; Coustau, Chevillon & ffrench‐Constant 2000; Gassmann, Onstad & Pittendrigh 2009). Fecundity costs apply irrespective of other factors such as competition; for any given set of conditions, resistant insects always produce fewer progeny than susceptible insects. Therefore, the resistance allele is slower to spread, more susceptible insects may be killed within each generation, and the fitness cost synergizes with the high‐dose/refuge strategy (Carrière & Tabashnik 2001; Tabashnik 2004; Alphey *et al*. 2008).

In contrast, the expression of costs to competition is contextual. Competition costs only impair the spread of the resistance allele when the population exceeds a threshold density beyond which resistant insects fail to compete and decline. Such a cost is more reflective of the weaker fitness costs reported in field‐evolved resistance to Bt crops (Jakka, Knight & Jurat‐Fuentes 2014; Dangal & Huang 2015; Ingber & Gassmann 2015). Competition costs were insufficient to delay the evolution of resistance under constant selection (Fig. S2) and provided only marginal benefits when the model was free to control the Bt exposure of the pest (Figs 3 and 5). When resistant insects were less competitive than susceptible insects, the decision model consistently allowed the total population to grow towards this threshold density before adopting a decision set that regulated the total population around this value (Figs 3 and 5). This maintains the population of susceptible insects within a range for which the further growth of the resistant population is blocked, allowing for a degree of killing without fixing the resistance allele. However, to generate sufficient numbers of susceptible insects that the reduced competitive ability of resistant insects becomes pertinent, extremely large refuges must be planted for much of the time horizon. While this requirement for a large refuges runs contrary to the desires of growers, who favour smaller refuges, these decision sets highlight that fitness differences between resistant and susceptible insects need not translate into management benefits. For fixed refuges, competition costs did not delay resistance due to the high rate of susceptible mortality; competition only becomes a limiting factor once the population is comprised of primarily resistant insects, and thus, the cost provides no hindrance. Similarly, fecundity costs only become limiting under fixed selection when refuges are large enough to enable the survival of a sufficient number of susceptible insects that they are able to outpace the growth of the resistant genotype.

Heterogeneous density dependence neither accelerated nor impeded the evolution of resistance when refuges were fixed (Fig. S1). High rates of toxin mortality mitigate any effect of competition upon the dynamics and genetics of the population. Under the dynamic programming model, heterogeneous density dependence influenced transient features of the decision set, but average model behaviour was unaffected (Figs 5 and S6, S7). Thus, spatial variation in the strength of density dependence as implemented here was not a significant factor in the evolution or management of resistance. However, if the model were to be extended to include additional behavioural and ecological idiosyncrasies such as larval dispersal or oviposition biases, then local variation in the intensity of competition could become significant. For example, female fall armyworm *Spodoptera frugiperda* preferentially oviposit on undamaged transgenic Bt crops in response to larval feeding on refuge plants which is predicted to accelerate the evolution of resistance (Téllez‐Rodríguez *et al*. 2014). Therefore, it remains significant that additional thought be given to the role of intraspecific (Okuyama & Hsu 2013) and interspecific (Becker & Liess 2015) interactions in shaping the evolution of resistance in the field.

Although the decision sets proposed by this model were generated for a simple agricultural system, a key implication of our results is that additional insect control which operates independently of Bt resistance genes, such as sterile insect releases (Tabashnik *et al*. 2010; Harvey‐Samuel *et al*. 2015), may be beneficial in prolonging susceptibility, suppressing pests and protecting yields (Alphey, Bonsall & Alphey 2009), potentially making smaller refuges optimal. Furthermore, we have assumed transgenic crops express a single toxin but pyramided crops expressing multiple toxins are now available (Tabashnik, Brévault & Carrière 2013) and may present an additional barrier to resistance by necessitating the occurrence of two distinct mutations. While the risk of cross resistance by major agricultural pests is non‐trivial, the possibility remains that, as supported by our results, the suppression of pest populations to preserve susceptibility to Bt crops may require larger refuges (Carrière, Fabrick & Tabashnik 2016).

The pleiotropic effects of resistance alleles may have a crucial role to play in the evolution of resistance to insecticidal toxins such as those expressed by Bt crops, contributing to both the rate of spread and management of resistance. If the fitness costs associated with resistance alleles do not consistently affect the survival or reproduction of resistant insects, then the disadvantageous effects of these genes can be masked by selection. Resistance may then evolve more rapidly than originally projected and prescribed refuges will not provide adequate defence, necessitating either a revision of refuge sizes or the incorporation of additional tactics to maintain effective suppression. Long‐term resistance management will benefit from an improved understanding of the interplay between the realization of fitness costs and the ecology of pests in the field.

## Supporting information


**Figure S1.** Time series for pest density and resistance allele frequency over 100 generations for uniform and heterogeneous density dependence.
**Figure S2.** Population density and resistance allele frequency for fixed refuge sizes and a 25% fitness penalty to competition.
**Figure S3.** As for Figure S2 but with larger initial population densities.
**Figure S4.** Time series of population density and resistance allele frequency for a 25% cost to fecundity and large fixed refuges (φ = 0·5, 0·2).
**Figure S5.** Time series for forward simulations with costless resistance and uniform density dependence.
**Figure S6.** As for Figure S5 but with heterogeneous density dependence.
**Figure S7.** Time series for forward simulations with fitness costs on fecundity and heterogeneous density dependence.
