# Supplementary material for: Type of fitness cost influences the rate of evolution of resistance to transgenic Bt crops
Source: J Appl Ecol. 2016 May 5;53(5):1391–401. doi: 10.1111/1365-2664.12680 (PMC5026168; doi:10.1111/1365-2664.12680)
Supplement: Supplementary file 1 — Figure S1. Time series for pest density and resistance allele frequency over 100 generations for uniform and heterogeneous density dependence. Figure S2. Population density and resistance allele frequency for fixed refuge sizes and a 25% fitness penalty to competition. Figure S3. As for Figure S2 but with larger initial population densities. Figure S4. Time series of population density and resistance allele frequency for a 25% cost to fecundity and large fixed refuges (φ = 0·5, 0·2). Figure S5. Time series for forward simulations with costless resistance and uniform density dependence. Figure S6. As for Figure S5 but with heterogeneous density dependence. Figure S7. Time series for forward simulations with fitness costs on fecundity and heterogeneous density dependence. [file JPE-53-1391-s001.docx]

**Supplementary Materials**


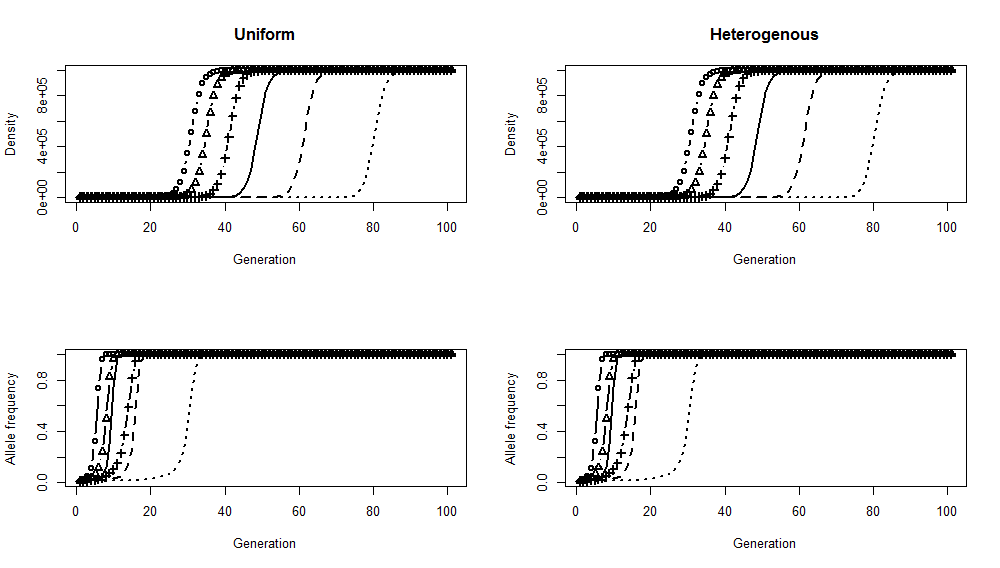


*Figure S1:* Time series of the change in pest density (top row) and resistance allele frequency (bottom row) over 100 generations of selection. In the left column, density dependent mortality acts uniformly across both the toxic and refuge patches while in the right column density dependent mortality is patch specific and scales inversely with patch size. Values are plotted for two levels of dominance, fully recessive resistance, $h=0$ (lines without points), and partially recessive resistance, $h=0.05$ (lines with points). Results are plotted for three Bt cropping regimes. Time series for a 5% refuge ($\varphi=0.95$) are depicted as solid lines ($h=0$) and lines with circular points ($h=0.05$). For a 10% refuge ($\varphi=0.9$) results are plotted as dashed lines ($h=0$) and lines with triangular points ($h=0.05$). For a 20% refuge results are plotted with dotted lines ($h=0$) and crosses ($h=0.05$). In all instances the population is founded by $N_{0}=1000$ insects with an initial resistance allele frequency of $q_{0}=0.01$.


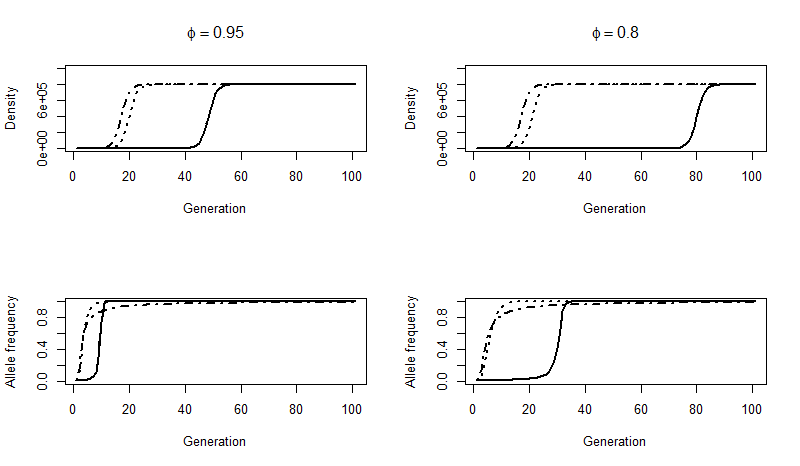


*Figure S2:* Population size (top row) and resistance allele frequency (bottom row) over 100 generations for landscapes planted with 95% (left column) and 80% (right) Bt respectively when resistance is associated with a 25% reduction in competitive ability ($\alpha_{rr}=1.25\alpha_{ss}$). Density dependence is uniform and undercompensating ($\beta=1$). Solid black lines refer to recessive resistance with a recessive fitness cost ${(h}_{res}=0, h_{cost}=0)$. Dashed lines depict recessive resistance with a dominant fitness cost${(h}_{res}=0, h_{cost}=1)$. Dotted lines refer to additive resistance with a dominant fitness cost $(h_{res}=0.5, h_{cost}=1)$ and lines with both dots and dashes illustrate dominant resistance with a dominant fitness cost $(h_{res}=1, h_{cost}=1)$. Populations were founded with $N_{0}=1000$ insects and an initial resistance allele frequency of $q_{0}=0.01$.


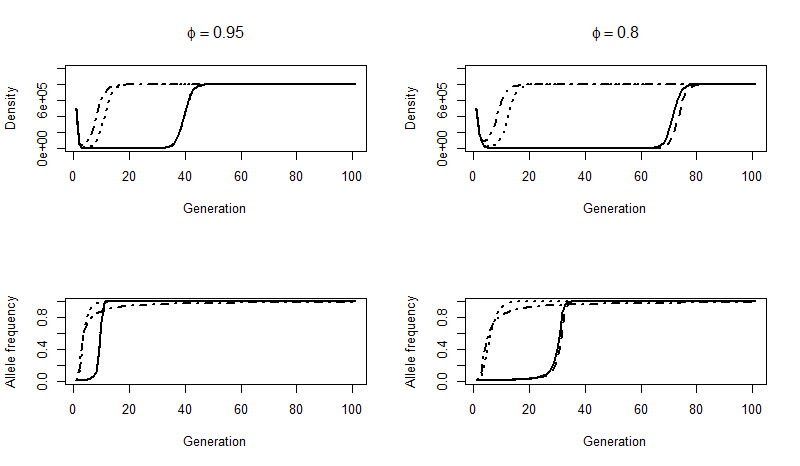


*Figure S3:* Population size (top row) and resistance allele frequency (bottom row) over 100 generations for landscapes planted with 95% (left column) and 80% (right column) Bt respectively when resistance is associated with a 25% reduction in competitive ability ($\alpha_{rr}=1.25\alpha_{ss}$). Density dependence is uniform and undercompensating ($\beta=1$). Solid black lines refer to recessive resistance with a recessive fitness cost ${(h}_{res}=0, h_{cost}=0)$. Dashed lines depict recessive resistance with a dominant fitness cost${(h}_{res}=0, h_{cost}=1)$. Dotted lines refer to additive resistance with a dominant fitness cost $(h_{res}=0.5, h_{cost}=1)$ and lines with both dots and dashes illustrate dominant resistance with a dominant fitness cost $(h_{res}=1, h_{cost}=1)$. Populations were founded with $N_{0}=500,000$ insects and an initial resistance allele frequency of $q_{0}=0.01$.


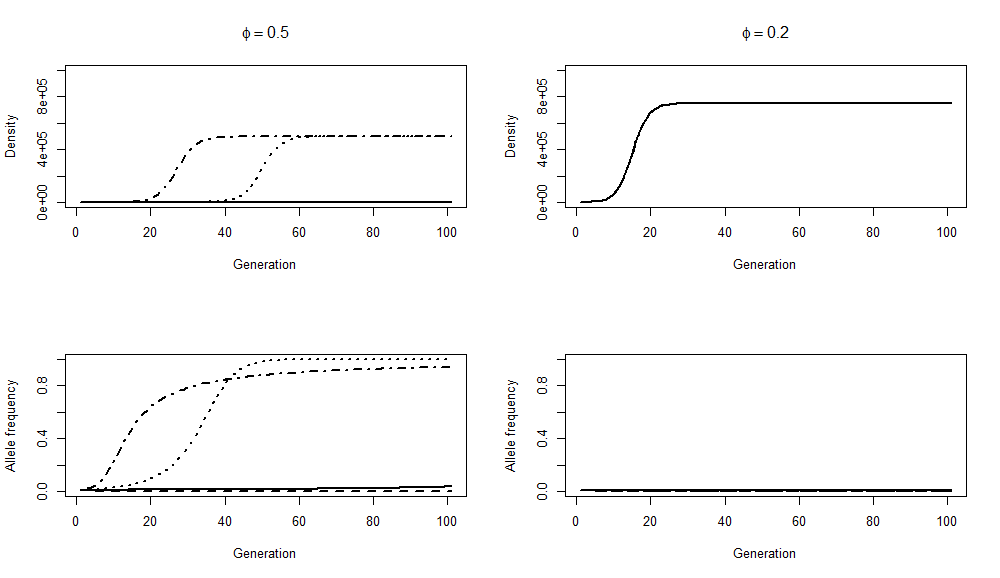


*Figure S4:* Population size (top row) and resistance allele frequency (bottom) over 100 generations for landscapes planted with 50% (left column) and 20% (right) Bt respectively when resistance is associated with a 25% reduction in fecundity ($\lambda_{rr}=0.75\lambda_{ss}$). Density dependence is uniform and undercompensating ($\beta=1$). Solid lines show recessive resistance with a recessive fitness cost ${(h}_{res}=0, h_{cost}=0)$. Dashed lines depict recessive resistance with a dominant fitness cost${(h}_{res}=0, h_{cost}=1)$. Dotted lines refer to additive resistance (co-dominant) with a dominant fitness cost $(h_{res}=0.5, h_{cost}=1)$ and dash-dot lines illustrate dominant resistance with a dominant fitness cost $(h_{res}=1, h_{cost}=1)$. Populations were founded with $N_{0}=1000$ insects and initial resistance allele frequency $q_{0}=0.01$


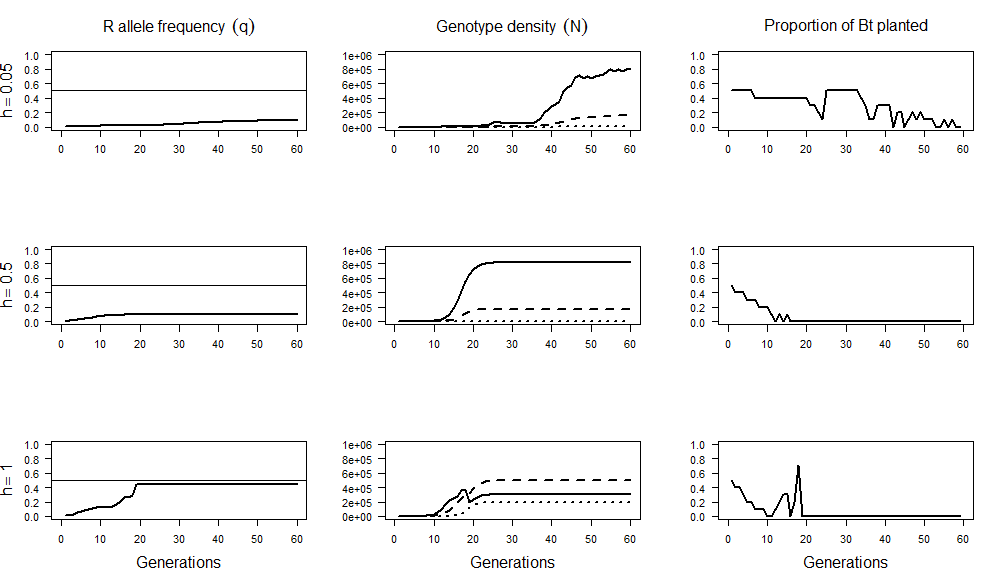


*Figure S5:* Resistance allele frequency (leftmost column), genotype abundance (central column) and proportional allocation of space to Bt crops over $T=60$ generations when resistance is cost free. Density dependence is uniform and undercompensating with $\beta=1$. The dominance of resistance was increased from $h=0.05$ (top row), to $h=0.5$ (central row) and $h=1$ (bottom row). The solid horizontal line on plots of resistance allele frequency indicates the critical allele frequency, $q_{c}=0.5$ beyond which resistance management is deemed to have failed. For plots of genotype specific density, solid lines represent susceptible homozygotes (ss), dashed lines represent heterozygotes (sr) and dotted lines represent resistant homozygotes (rr). Populations were founded by $N_{0}=$ $1000$ insects with a resistance allele frequency of $q_{0}=0.01$.


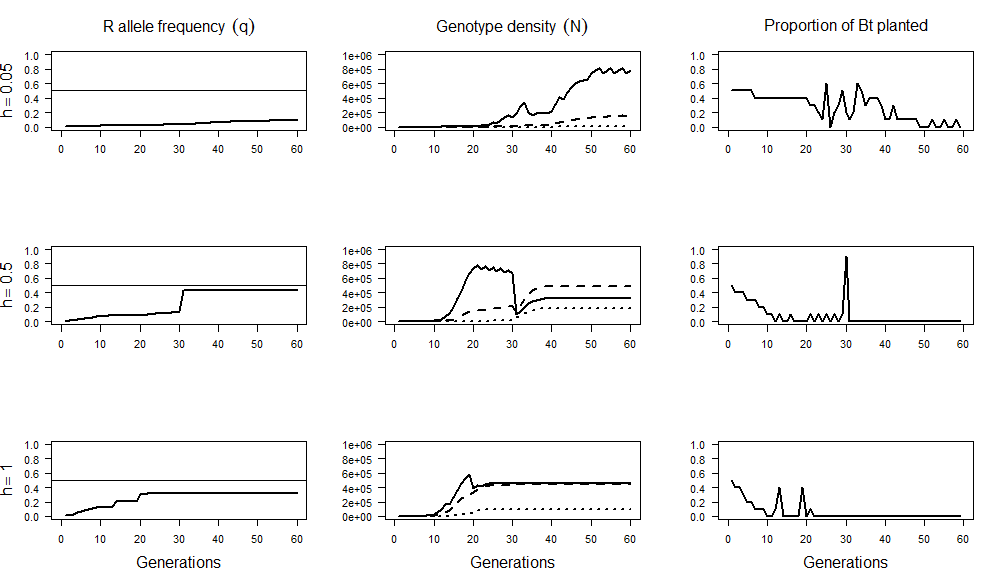


*Figure S6:* Resistance allele frequency (leftmost column), genotype abundance (central), and the proportional allocation of space to Bt crops over $T=60$ generations when resistance is cost free. Density dependence is heterogeneous and undercompensating ($\beta=1$). The dominance of resistance was increased from $h=0.05$ (top row), to $h=0.5$ (central row) and $h=1$ (bottom row). The solid horizontal line on plots of resistance allele frequency indicates the critical allele frequency, $q_{c}=0.5$ beyond which resistance management is deemed to have failed. For plots of genotype specific density, solid lines represent susceptible homozygotes (ss), dashed lines represent heterozygotes (sr) and dotted lines represent resistant homozygotes (rr). Populations were founded by $N_{0}=$ $1000$ insects with a resistance allele frequency of $q_{0}=0.01$.


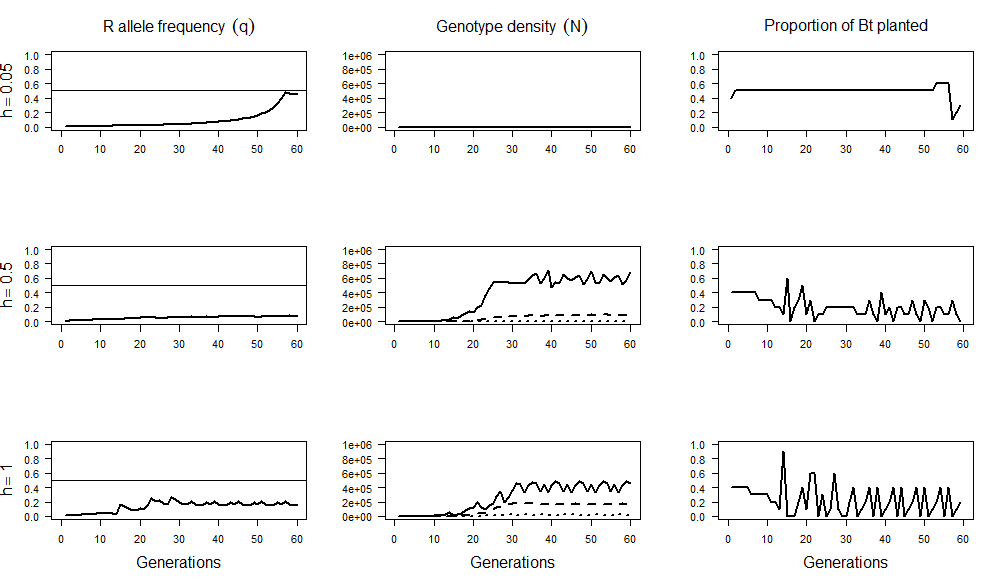


*Figure S7:* Resistance allele frequency (leftmost column), genotype abundance (central) and the proportional allocation of space to Bt crops over $T=60$ generations when resistance is associated with a 25% reduction in fecundity ($\lambda_{rr}=0.75\lambda_{ss}$). Density dependence is heterogeneous and undercompensating. The dominance of resistance was increased from $h=0.05$ (top row), to $h=0.5$ (central row) and $h=1$ (bottom row). The solid horizontal line on plots of resistance allele frequency indicates the critical allele frequency, $q_{c}=0.5$ beyond which resistance management is deemed to have failed. For plots of genotype specific density, solid lines represent susceptible homozygotes (ss), dashed lines represent heterozygotes (sr) and dotted lines represent resistant homozygotes (rr). Populations were founded by $N_{0}=$ $1000$ insects with a resistance allele frequency of $q_{0}=0.01$
